# Social inequality in the association between life transitions into adulthood and depressed mood: a 27-year longitudinal study

**DOI:** 10.3389/fpubh.2024.1286554

**Published:** 2024-02-27

**Authors:** Magnus Jørgensen, Otto R. F. Smith, Bente Wold, Ellen Haug

**Affiliations:** ^1^Department of Health Promotion and Development, Faculty of Psychology, University of Bergen, Bergen, Hordaland, Norway; ^2^Department of Health Promotion, Division of Mental and Physical Health, Norwegian Institute of Public Health (NIPH), Bergen, Hordaland, Norway; ^3^Department of Teacher Education, NLA University College, Bergen, Hordaland, Norway

**Keywords:** life transitions, life course, depressed mood, socioeconomic status, the adolescent pathway model, cohabitation, moving out, employment

## Abstract

**Background:**

Few studies have considered the life-course development of depressive symptoms in relation to life transitions in early-adulthood and whether these might affect depressive trajectories differently depending on specific indicators of parental socioeconomic status (SES). In the present work, we explore these questions using the adolescent pathway model as a guiding framework to test socially differential exposure, tracking and vulnerability of the effects of life transitions on depressed mood across different socioeconomic backgrounds.

**Methods:**

Latent growth modeling was used to estimate the associations between indicators of parental SES (parental education and household income) and depressed mood from age 13 to 40 with life transitions (leaving the parental home, leaving the educational system, beginning cohabitation, attaining employment) as pathways between the two. Our analyses were based on a 27-year longitudinal dataset (*n* = 1242) of a Norwegian cohort with 10 time points in total. To make socioeconomic comparisons, three groups (low, mid, and high) were made for parental education and income respectively.

**Results:**

Depressed mood decreased from age 13 to 40. The low and high parental education groups showed a stable difference in depressed mood during early adolescence, which decreased in young adulthood and then increased slightly in mid-adulthood. The low household income group showed higher depressed mood across young adulthood compared to the medium and higher household income groups. For life transitions, leaving the parental home and beginning cohabitation was associated with an added downturn of the trajectory of depressed mood when adjusting for other transitions. However, adolescents with high parental education showed a relatively stronger decrease in depressed mood when leaving the parental home. Similarly, adolescents with a high household income showed a relatively stronger decrease in depressed mood when leaving the educational system.

**Conclusions:**

Depressed mood decreased over time and developed differently depending on parental education and household income. Life transitions were generally associated with reductions in depressed mood across time, but lower SES youths were not found to be more socially vulnerable these effects.

## 1 Introduction

Adult depression is a leading cause of disability worldwide and is often preceded by subclinical depression in adolescent years (1–5). Depressed mood is a suitable measure in this respect as it retains key aspects of depression, but without including some of the more ambiguous symptoms (i.e., fatigue, changes in appetite and weight, physical pains, anhedonia etc.) (6). During adolescence, rapid psychosocial maturation and brain development occurs, making adolescents more sensitive to stressful environments and life changes (7–9). This seems especially true for adolescents in disadvantaged households (10). Accordingly, a large body of research has documented how parental socioeconomic status (SES) is intimately linked to adolescent health outcomes (11, 12). Several studies also report that lower parental SES is associated with higher depressive outcomes in offspring that persist into adulthood (13–27). This is also the case for depressive trajectories from adolescence and into adulthood. For example, in a US longitudinal study, it was found that early cumulative socioeconomic adversity significantly predicted elevated levels of depressive symptoms, but also a greater decline in depressive symptoms from adolescence to young adulthood (26). Similarly, another longitudinal study also found that lower family income predicted a trajectory of stable high levels of depressive symptoms from early adolescence to young adulthood (25). These findings lend support to the Adolescent Pathway Model (APM), which suggests that adolescent socioeconomic background shapes depressive outcomes throughout the life course (28). More specifically, the APM propose that parental SES results in different levels of depressive symptoms (socially differential exposure) that develop differently over time (socially differential tracking) with increased long-term vulnerability through a number of different pathways for lower SES youths (socially differential vulnerability).

But, as noted by several scholars, developmental discontinuities in depressive trajectories can occur because of major educational and social transitions during the transition into adulthood (14, 29, 30), and could be a pathway through which parental SES impacts the development of depressed mood. According to the life course literature, four of the major life transitions that usually occur during the transition into adulthood are: Leaving the parental home, leaving the educational system, beginning cohabitation with a romantic partner, and attaining full-time employment (31). These transitions are major, because they bring about new demands and opportunities, and earlier studies indicate that failing to solve these is associated with decreased wellbeing, happiness, and life satisfaction (32–37). This has led scholars to gain a renewed interest in life transitions as pathways from adolescence to adult depressive outcomes (2, 3, 28, 38–40). However, the literature is scarce, and the effects of life transitions on long-term depressive trajectories have received little empirical attention in relation to socially vulnerable adolescents in existing studies (7, 8, 14, 15, 28) - evident by a lack of focus on socioeconomic background.

Still, some earlier research indicates that disadvantaged adolescents might show socially differential susceptibility to the effects of several early life transitions. For example, leaving the parental home might have more positive effects on depressed mood for lower SES youths with studies indicating that lower SES youth more often grow up in troubled neighborhoods and chaotic households with frequent family conflicts (41). In a similar vein, lower parental SES has been associated with a higher likelihood of adolescents beginning early cohabitation with a partner which could reflect the need to get away from conflicts in the household (42). On the other hand, independent living might also incur greater costs and psychological pressure on disadvantaged youth as they tend to receive less financial support from home (43, 44). It is also possible that disadvantaged youths fare better off when leaving the educational system as they often experience high levels of peer problems, academic failures, and adjustment issues in the school system (45, 46). For instance, in a Chinese study peer problems and academic performance were significant mediators of the effects of family SES on internalizing problem behavior (45). Similarly, entering full-time employment during emerging adulthood could have substantial effects on depressive outcomes. A systematic review by van der Noordt, Ijzelenberg (47) report that employment is a strong protective factor against developing depression and mental illness across both age groups and genders. Still, this might not hold true for lower SES youths who often struggle finding decent and stable employment upon transitioning from higher education (48). Together, these findings point to the need for more research to test if life transitions could play a critical role in shaping long-term social disparities in adult depressive outcomes. Furthermore, research in this area could aid interventions seeking to bolster against the development of clinical depression (49). But, to achieve this aim, research is also needed that clearly distinguishes between different indicators of parental SES as earlier studies indicate that these are not interchangeable in their effects and presumed causes (50). Parental income exerts direct effects on housing, diet quality and amount of financial support offspring receive during their adult development (e.g., financing their first home, paying new expenses associated with independent living etc.) (51). In contrast, parental education is more stable than income and is believed to be associated with the transmission of knowledge and skills that can positively affect offspring's ability to cope with stressful life changes as well as their ability to navigate societal institutions and bureaucracies (e.g., accessing health care, taking care of one's health etc.) (51). In the present study, we thus address the following research questions: (1) How does depressed mood develop from adolescence to adulthood (i.e., pathway I in the APM) and are parental education and income in adolescence associated with differential exposure and development of depressed mood from adolescence to adulthood? (i.e., mechanism A and C in the APM), (2) Do common early life transitions (leaving the parental home, beginning cohabitation, leaving the educational system, and attaining full-time employment) affect this development? (i.e., pathway IV in the APM), (3) Do life transitions show differential vulnerability effects on adult depressed mood based on parental education and household income in adolescence? (i.e., mechanism D in the APM). In investigating these questions, we employ a novel approach with several substantial contributions to current research on life transitions. Firstly, we test multiple life transitions in combination. Secondly, we test these transitions in the context of adolescent socioeconomic background while also distinguishing between the effects of individual indicators of parental SES, and thirdly, we cover an extensive 27-years from age 13 to 40.

## 2 Methods

### 2.1 Participants

The present study used data from the Norwegian Longitudinal Health Behavior Study (NLHB) (52). NLHB is a longitudinal dataset following a cohort of Norwegian adolescents (*n* = 1242) from age 13 in 1990 to age 40 in 2017 - totaling ten survey waves (1990, 1991, 1992, 1993, 1995, 1996, 1998, 2000, 2007, and 2017, see Table 1 for response rates). The sample was extracted from 22 junior high schools in the former Hordaland County. New students were invited to participate during the first few waves of data collection bringing the total sample size up to 1242 unique individuals. Hordaland County is often considered one of the most representative counties in Norway (i.e., close to the national average) as based on household disposable income per capita (53), educational distribution (54) and general life satisfaction (55). However, notably the NLHB started data collection at a time when the population in Hordaland was more ethnically homogenous - limiting the external validity in this respect.

**Table 1:** Response rate and characteristics for the measure of depressed mood.

**Measurement occasion**	**Participant response rate^1^**	**Share of responders providing complete item-level data**	**Mean and standard deviation**
**Year (age)**	**% (** ***n*** **)**	**%**	**M (SD)**
1990 (13)	58.1 (722)	92.0	2.27 (0.90)
1991 (14)	75.8 (941)	98.4	2.19 (1.01)
1992 (15)	72.5 (900)	95.4	2.41 (1.08)
1993 (16)	56.8 (706)	97.5	2.15 (1.04)
1995 (18)	62.4 (775)	99.4	2.38 (1.08)
1996 (19)	51.0 (633)	98.9	2.17 (1.07)
1998 (21)	47.3 (588)	96.1	1.99 (0.98)
2000 (23)	50.6 (629)	99.1	1.89 (0.96)
2007 (30)	42.8 (532)	99.1	1.73 (0.85)
2017 (40)	36.2 (449)	95.8	1.63 (0.83)

^1^N_*total*_ = 1242.

The NLHB dataset focuses mainly on variables pertaining to perceived health and health-related behaviors. When adolescents were aged 13, 16 and 19 (1990, 1993, and 1996) participants' the parents (*n* = 948, *n* = 600, and *n* = 622) were also surveyed on relevant variables, such as educational attainment and household income. Of the 1,242 originally included participants, 1,099 (88.5%) provided valid data on at least one measurement occasion for the main outcome variable, depressed mood, which is therefore used as the real sample size for the current study. Attrition analyses on key variables are reported in Appendix A. The NLHB data collection was approved by the Data Inspectorate of Norway and a recommendation was received from the Regional Committee of Medical Research Ethics (REK). Written consent was obtained from all participants. Detailed information on the NLHB dataset is provided elsewhere (56, 57).

### 2.2 Outcome variable

Depressed mood was measured at all time points using an adapted version of the Depressive Tendencies Scale developed by Alsaker (58). In alignment with Holsen et al. (56), we use this scale to measure depressed mood due to the high overlap with this construct. The adapted scale contains seven items with a 6-point Likert Scale ranging from “applies exactly” to “does not apply at all.” Examples of items include: “Sometimes I think everything is so hopeless that I don't feel like doing anything,” “I am often sad without seeing any reason for it.” And “I think my life is mostly miserable.” Two items pertaining to suicidality were excluded from the original nine-item scale due to ethical concerns about triggering suicidal thoughts in vulnerable respondents and not having resources to follow up on distressing reactions. To the authors' knowledge, this scale has not been validated against similar measures in other studies. However, an analysis with latent variables showed a correlation of 0.82 with the Center for Epidemiological Studies Depression Scale (CES-D) in the NLHB 1996 dataset (56). Thus, there is evidence for concurrent validity. Further, the Depressive Tendencies Scale was reliable across all time points as indicated by Cronbach alpha coefficients ranging from 0.82 at age 13 (1990) to 0.94 at age 40 (2017).

We used mean sum scores for depression to make our models more parsimonious and to ease parameter estimation. Occasion-specific mean sum scores were calculated for cases with responses on a minimum of four items. Given high Cronbach alpha values and the fact that most respondents provided complete item-level data, we anticipated that this approach would only minimally affect the estimates of interest in this study (See also Table 1). As depressed mood was assessed across a relatively large time span, we tested for measurement invariance and found support for partial scalar measurement invariance across time suggesting that valid comparisons of mean differences across time can be made (see Appendix B).

### 2.3 Early life transitions

All transition variables were created in SPSS 26. These are: Leaving the parental home, leaving the educational system, beginning cohabitation, and attaining employment (See Table 2). Only time points with data available for a given life transition are shown in Table 2. A blank cell thus indicates that no data was available for a particular life transition at a particular time point. Time points for ages 30 and 40 (2007 and 2017) are not shown in Table 2 as these were intended as control variables. Parenthood was also used as a control variable at every time point and is thus not shown in Table 2. Leaving the parental home and beginning cohabitation was measured by asking participants: “Who are you living with at the moment?” at ages 13, 18, 19, 21, 30, and 40. Leaving the parental home was coded as 0 = Living with parents and 1 = Other living situation. Beginning cohabitation was coded as 0 = Other living situation and 1 = Living with spouse/partner. Each living situation was measured as a binary yes/no variable. The response categories were: “Living with mom,” “Living with dad,” “Living with mom's cohabitant/spouse,” “Living with dad's cohabitant/spouse,” “Moving back and forth between mom and dad,” “Living with foster parents,” “Living with others,” “Living alone,” “Living with parents,” “Living with friends” and “Living with cohabitant/spouse.” Leaving the educational system and attaining employment was measured by asking participants at age 16: “What are you currently doing?” and “What is your current occupation?” from age 18 to 40. At age 13, all participants were assumed to be students. At age 16, response categories were “High school,” “Vocational school,” “10th grade,” “Working” and “Other.” From age 18 to 40 response categories were: “Student,” “Working (minimum 30 h per week),” “Working part time (< 30 h a week),” “Unemployed,” “In military/civil services,” “Household work,” “On leave” and “Other.” Leaving the educational system was coded as 0 = student and 1 = non-student. Attaining employment was coded as 0 = unemployed and 1 = employed. Participants studying and working part-time were coded as students while participants working full-time, and studying were coded as employed. Return to pre-transition state were coded as missing for all consecutive survey years as few returns were present for all life transitions. Lastly, phi coefficients between life transitions were examined for collinearity issues.

**Table 2:** Descriptive data on life transitions.

	**Leaving the parental home**	**Leaving the educational system**	**Beginning Cohabitation**	**Attaining employment**
**Age 16**				
Complete transition % (*n*)		4.3 (47)		
No transition % (*n*)		60.0 (659)		
Missingness % (*n*)		35.8 (393)		
**Age 18**				
Complete transition % (*n*)	5.1 (56)	5.2 (57)		5.8 (64)
No transition % (*n*)	48.6 (534)	46.0 (505)		64.9 (713)
Missingness % (*n*)	53.7 (509)	48.9 (537)		29.3 (322)
**Age 19**				
Complete transition % (*n*)	18.4 (202)	16.3 (179)	5.4 (59)	10.9 (120)
No transition % (*n*)	33.5 (368)	34.2 (376)	53.0 (582)	41.9 (460)
Missingness % (*n*)	48.1 (529)	49.5 (544)	41.7 (458)	47.2 (519)
**Age 21**				
Complete transition% (*n*)	13.1 (144)	5.8 (64)	6.6 (72)	6.0 (66)
No transition % (*n*)	28.8 (316)	30.0 (330)	38.5 (423)	33.3 (366)
Missingness % (*n*)	58.1 (639)	64.1 (705)	55.0 (604)	60.7 (667)
**Age 23**				
Complete transition % (*n*)	7.5 (82)	7.5 (82)	9.6 (106)	7.6 (84)
No transition % (*n*)	31.2 (343)	24.9 (274)	31.1 (344)	27.7 (304)
Missingness % (*n*)	61.3 (674)	67.6 (743)	59.1 (649)	64.7 (711)

Participants who already completed a transition were coded as missing. Percentages calculated based on the analytic sample of n = 1099.

### 2.4 Covariates

Gender and parental SES were used as time-invariant covariates, whereas parenthood (at ages 19, 21, 23, 30, and 40), cohabitation (at ages 30 and 40), and attaining employment (at ages 30 and 40) were used as time-varying covariates. *Gender* was reported at age 13 as either male (54.4%) or female (45.6%). Educational level and household income were used as indicators of parental SES. Parents' report of pretax household income in 1995 was reported in 1996 using one of six categories. Using the 01–01–1995 NOK-EURO exchange rate, this corresponds to “Less than NOK 100.000 (~ € 11 899),” “NOK 100–199.000 (~ € 11 900–23 999),” “NOK 200–299.000 (~ € 24 000–34 499),” “NOK 300–399.000 (~ € 35 500–46 399),” “NOK 400–499.000 (~ € 47 400–59 299)” and “NOK 500.000 or more (~ € 59 300 or more).” Parental education was reported in 1996 on a 6-point scale from “0 years of education after elementary school,” “1–2 years of education after elementary school,” “3 years of education after elementary school,” “ <4 years at university/college,” “More than 4 years at university/college” and “Other.” Missing values and the last category “other” were replaced with adolescents' reports of parental educational attainment in one of six categories: “Elementary school (6 years, ages 7–12),” “Upper elementary school (3 years, ages 13–15),” “Upper secondary school (Vocational)” (ages 16–18), “Upper secondary school (Office/trade) (ages 16–18),” “Upper secondary school (General studies) (ages 16–18)” and “University/higher education (from age 19).” Information on parental educational level was available for *n* = 968, whereas information on parental household income was available for *n* = 615. To ease interpretation of the study results, the parental educational and income variables were recoded into one of three categories to reflect low, medium, and high levels of these two variables. For parental educational level 26.5% were classified as low, 53.8% as medium, and 19.6% as high. For parental income level, 29.8% were classified as low, 47.6% as medium, and 22.6% as high. *Parenthood* was included as a time-varying covariate as very few participants had children before the age of 30. It was measured at ages 19, 21, 23, 30, and 40 by asking participants: “Do you have children?” (Yes/No). That is depressed mood at age 19 was regressed on parenthood at age 19, depressed mood at age 21 was regressed on parenthood at age 21, and so on. Lastly, *cohabitation and being employ*ed at age 30 and 40 were also added as time-varying covariates, at ages 30 and 40 because of the time gaps with the previous time points (age 23 to 30 and age 30 to 40) were too wide to consider these as life transitions.

### 2.5 Statistical analyses

**Figure 1:**
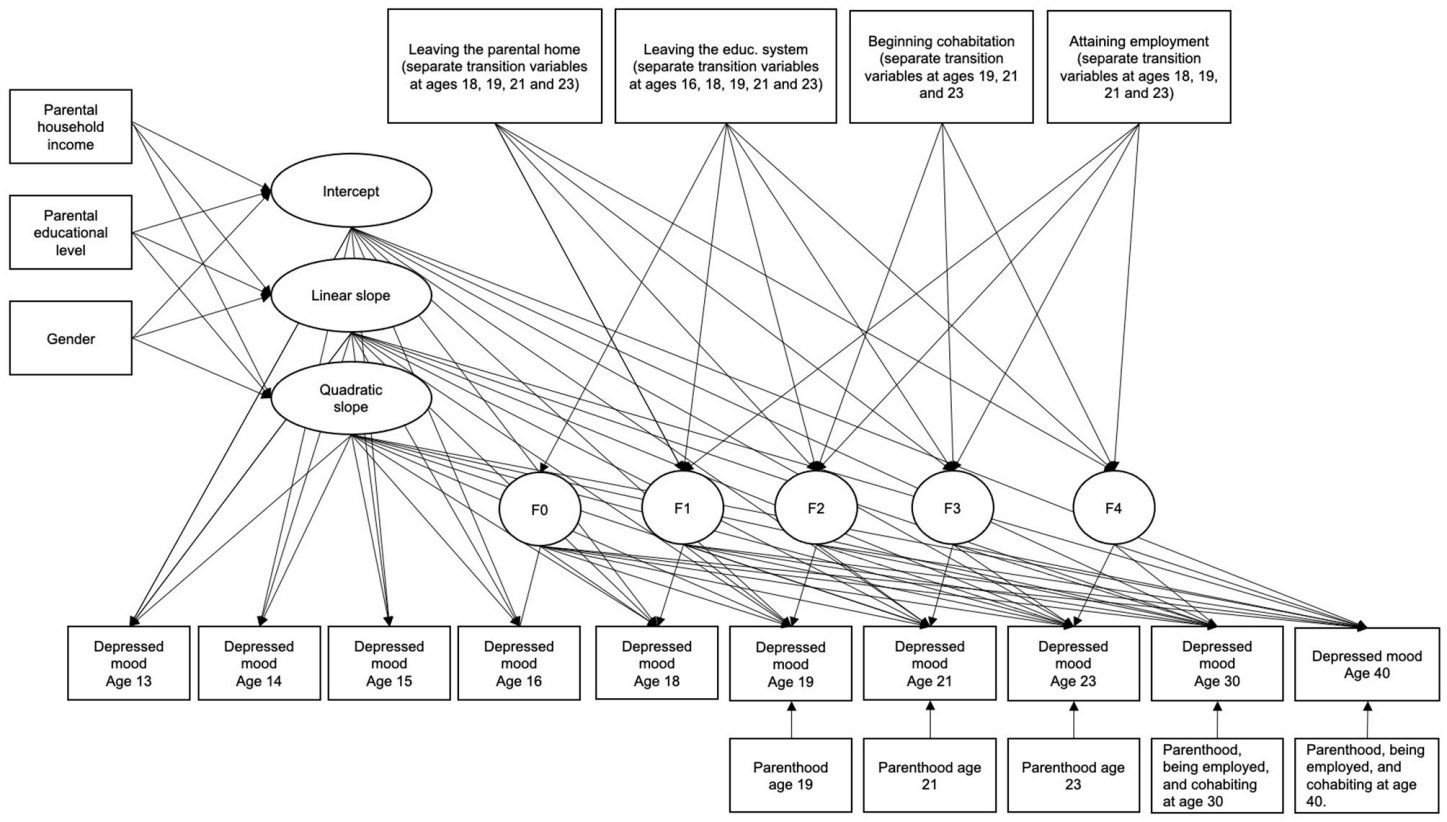
Conditional model with life transitions. The latent factors F0-F4 represent random intercepts for depressed mood from a particular time point to the last (e.g., from age 13 to 40, from age 14 to 40 etc.). Thus, a positive effect of a life transition on the random intercept would indicate a permanent improvement in depressed mood from that time point and onwards, whereas a negative effect of a life transition would indicate permanent deterioration in depressed mood from that time point and onwards. The growth slopes are not affected by the life transitions in this model.

We modeled the development of depressed mood using latent growth modeling (LGM) in Mplus (v. 8.7). Parameters were estimated using full maximum likelihood estimation (FIML). Exogenous variables were brought into the model as dependent variables to avoid list-wise deletion and to ensure that all participants with valid outcome data on at least one measurement occasion were included in the analyses (*n* = 1099). Model fit was evaluated primarily using the root mean squared error of approximation (RMSEA) and the Comparative Fit Index (CFI). Cut-off scores used were <0.05 for RMSEA and >0.90 for CFI (59, 60). Before conducting the primary analyses, we tested for measurement invariance using recommended model fit criteria by Chen (61) (See Appendix B). In the primary analyses, we build the models in steps of increasing complexity. First, we tested an unconditional growth model to estimate the growth factors (intercept and linear and quadratic slope). Secondly, we conditioned the growth model so that gender and the parental SES indicators predicted the growth factors. Thirdly, we added and tested early life transition effects by means of additional random intercepts with variance constrained to zero that were regressed on the transition variables (Figure 1). As such, the regression coefficient associated with the random intercept represents an increase or decrease in depressed mood at the time of a particular transition that persists over time (62). For each specific transition, we compared those who went through the transition with those who didn't, either because they didn't transition or because they stayed in the transitioned state. For life transitions that occurred at the same time point, their effects are mutually adjusted since they are associated with the same additional intercept. Wald tests were used to examine whether life transition effects at different time-points were time-invariant (e.g., does beginning cohabitation have the same effect on depressed mood when the transition occurs as age 16, 18, 19, 21, and 23 etc.). A significant Wald test was interpreted as evidence for a varying life transition effect across time points. We first tested each life transition individually (one life transition at a time) and then in conjunction with the other life transitions (all together). We also controlled for parenthood as a predictor of depressed mood at all time points in addition to beginning cohabitation and attaining employment at ages 30 and 40. These control variables accounted for some the variance in depressed mood to better estimate the unique effects of the early life transitions on the depressed mood trajectory. Data on leaving the parental home and leaving the educational system were not available for ages 30 and 40 (and were also judged as irrelevant for these ages). A multiple group model was used to test whether the parental educational level (low, medium, and high) and parental household income (low, medium, high) moderated the associations between early life transitions and depressed mood.

## 3 Results

### 3.1 Trajectory of depressed mood with age

Unconditional growth models of depressed mood from age 13 to 40 were tested with a linear slope (RMSEA = 0.089 CFI = 0.075) and in conjunction with a quadratic slope (RMSEA = 0.078, CFI = 0.817). Guided by modification indices, model fit was further improved by adding five first-order serial correlations, four second-order serial correlations, and one third-order serial correlation between the years 1991 and 2000. This yielded an acceptable overall fit (χ^2^ = 171.486, df = 36, *p* < 0.001, RMSEA = 0.059, 90% CI [0.050, 0.067], CFI = 0.920, SRMR = 0.065) with intercept *B* = 2.31 (95% CI = [2.26, 2.37], *p* < 0.001, variance = 0.53), linear slope *B* = −0.38 (95% CI = [−0.49, −0.28], *p* < 0.001, variance = 0.99) and quadratic slope *B* = 0.05 (95% CI = [0.01, 0.09], *p* < 0.001, variance = 0.09). The level of depressed mood decreased with age although, the rate of change per unit of time diminished somewhat during the study period. The estimated standardized mean change in depressed mood between 1990 and 2017 was 0.70, which suggests a clinically meaningful decrease in depressed mood over time.

### 3.2 Associations between depressed mood, life transitions, SES, and gender

**Figure 2:**
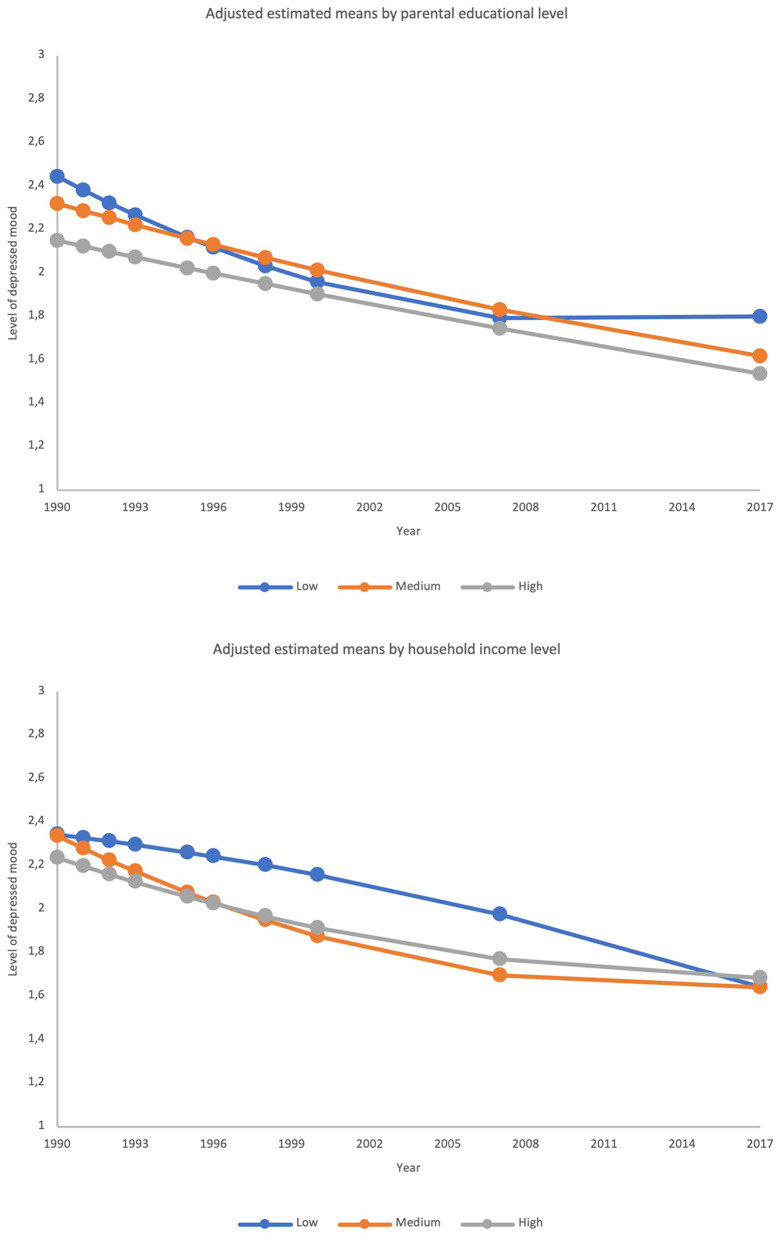
Adjusted estimated means of depressed mood by SES indicators. The upper graph shows means by parental education groups and the lower graph shows means by household income groups.

First, we conditioned the growth model on the main effects of gender, parental educational level, and parental household income. Firstly, the middle groups of the SES indicators were used as reference categories. The model gave acceptable fit indices (χ^2^ = 242.746, df = 71, *p* < 0.001, RMSEA = 0.047, 90% CI [0.041, 0.053], CFI = 0.920, SRMR = 0.049). Gender was only significantly associated with the intercept, but not with any of the growth parameters. Girls reported stable higher levels of depressed mood over-time (*B* = 0.27, 95% CI = [0.16, 0.38], *p* < 0.001). High parental educational level was associated with lower depressed mood at baseline (*B* = −0.17, 95% CI = [−0.31, −0.03], *p* = 0.018). Low parental educational level was associated with a lower *linear* slope (*B* = −0.30, 95% CI = [−0.58, −0.02], *p* = 0.038) and a higher *quadratic* slope (*B* = 0.12, 95% CI = [0.02, 0.22], *p* = 0.018) compared to medium parental educational level, whereas low parental household income was associated with a higher *linear* slope (*B* = 0.44, 95% CI = [0.11, 0.76], *p* = 0.008) and a lower *quadratic* slope (*B* = −0.16, 95% CI = [−0.28, −0.05], *p* = 0.004) compared to medium parental household income. For parental educational level, this seems to suggest that differences in depressed mood between the medium and high groups were relatively stable over time, whereas the difference between the low and medium groups decreased during the first part of the included life span and increased again during the second part of the included life span. For household income, the differences in depressed mood between the medium and high income groups were also relatively stable of time, whereas the differences between the low and medium income groups seem to increase during the first part of the included life span, and decreased again during the second part of the included life span (see also Figure 2).

Using the high SES groups as references, we found that the low parental education predicted a higher intercept (*B* = 0.29, 95% CI = [0.11, 0.48], *p* = 0.001) and steeper linear slope (*B* = −0.37, 95% CI = [−0.69, −0.05], *p* = 0.022) as well as a more positive quadratic slope (*B* = 0.13, 95% CI = [0.03, 0.24], *p* = 0.016) compared to high parental education. Low household income showed no difference in intercept (*B* = 0.11, 95% CI = [−0.11, 0.32], *p* = 0.337), linear slope (*B* = 0.25, 95% CI = [−0.12, 0.63], *p* = 0.190) or quadratic slope (*B* = −0.11, 95% CI = [−0.24, 0.02], *p* = 0.084). These findings suggest that substantial difference in depressed mood between low and high parental education which decreases near mid-adulthood. For household income, no differences between the low and high group emerged.

Building further upon the conditional growth model, we added each life transitions separately without adjusting for the effects of the other life transitions, but adjusting for parenthood (age 19, 21, 23, 30, and 40) as well as cohabitation and attaining employment at age 30 and 40. Wald tests indicated that life transitions did not differ across age (*p* > 0.05). We found a statistically significant effect for leaving the parental home (*B* = −0.21, 95% CI = [−0.29, −0.13], *p* < 0.001), beginning cohabitation (*B* = −0.23, 95% CI = [−0.33, −0.14], *p* < 0.001), attaining employment (*B* = −0.13, 95% CI = [−0.22, −0.04], *p* = 0.006) and leaving the educational system (*B* = −0.09, 95% CI = [−0.18, −0.01], *p* = 0.033).

When adding all life transitions to the conditional growth model (χ^2^ = 541.519, df = 308, *p* < 0.001, RMSEA = 0.026, 90% CI [0.023, 0.030], CFI = 0.908, SRMR = 0.042), see Figure 1), we found that only leaving the parental home (*B* = −0.16, 95% CI = [−0.25, −0.08], *p* < 0.001) and beginning cohabitation (*B* = −0.14, 95% CI = [−0.24, −0.03], *p* = 0.009) remained significantly associated with decreases in depressed mood over time. Leaving the educational system (*B* = −0.03, 95% CI = [−0.16, −0.10], *p* = 0.658) and attaining employment (*B* = −0.05, 95% CI = [−0.20, 0.10], *p* = 0.516) were not associated with changes in depressed mood. Again, life transitions did not differ across age according to Wald test results (all *p* > 0.05). The standardized effect size was 0.18 for leaving the parental home and 0.15 for beginning cohabitation - indicating small effect sizes overall (63).

**Figure 3:**
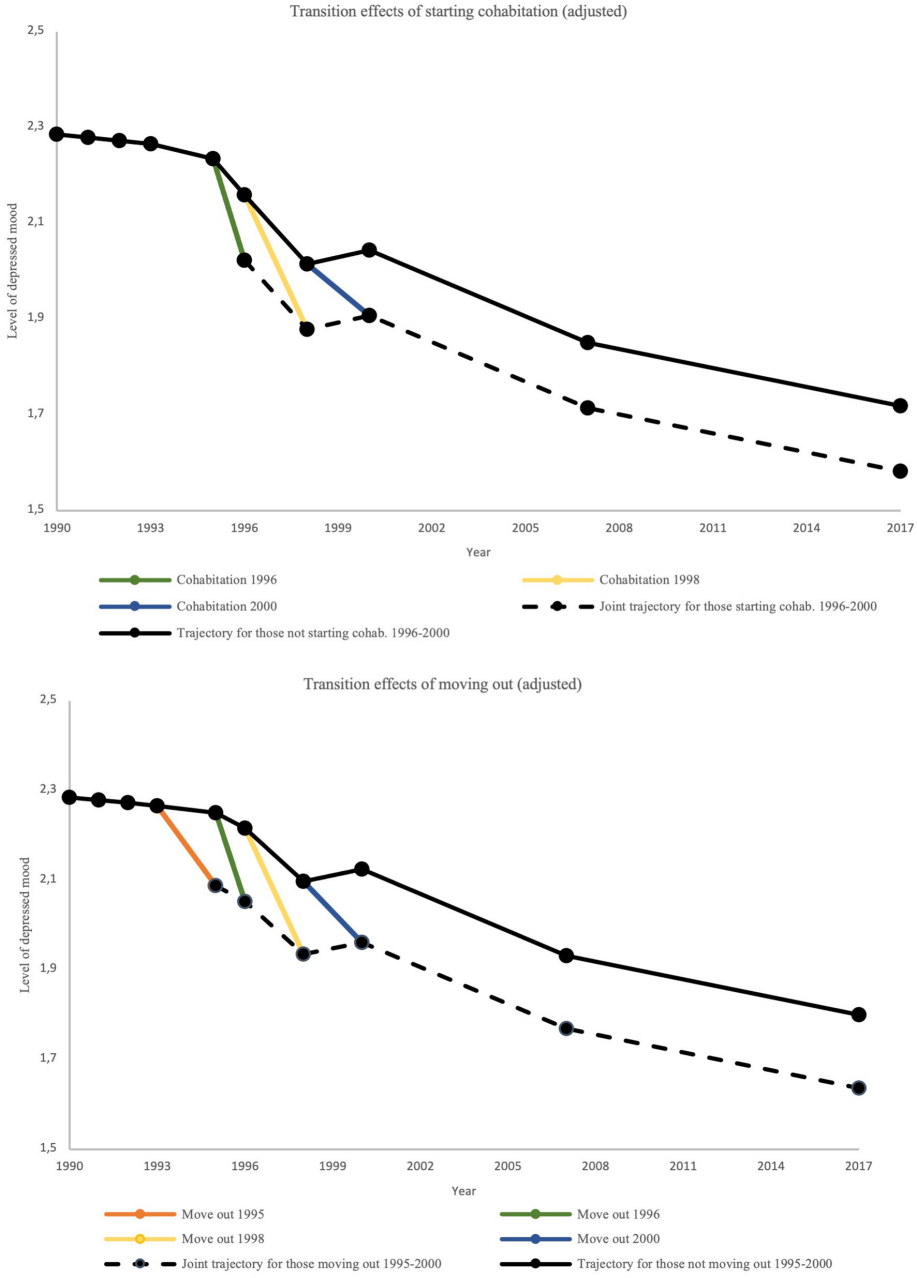
Estimated adjusted transition effects of moving out and cohabitation on depressed mood.

For those who moved out at age 18 (1995), the change in intercept was thus −0.16 points in depressed mood. This aligns with the sudden drop from the black line in Figure 3 in 1995 (orange line). This group subsequently follows the same trajectory shape as those not moving out, but at a level that is consistently −0.16 points lower (dashed black line). The same drop is observed for those moving out at age 19 in 1996 (green), age 21 in 1998 (yellow) and age 23 in 2000 (blue). A similar interpretation applies to those starting cohabitation from age 19 to 23 (1996–2000).

As a follow-up analysis, we re-run the model displayed in Figure 1 and checked to what extent leaving the parental home and beginning cohabitation were associated with the parental SES indicators, the intercept growth parameter, and the depression score at the measurement occasion prior to the actual transition (anticipatory effect) (62). To run this analysis, we had to use the original parental educational level (range 1–5) and household income (range 1–6) variables and treated them as continuous. Participants who moved out from age 18 to 23 (1995–2000) were on average from families with a somewhat higher educational level (*B* = 0.74, *p* = 0.001). There was no statistically significant association with parental household income and depressed mood at baseline or at the occasion prior to the actual transition. Participants who started cohabiting from age 19 to 23 (1996–2000) were from families with somewhat lower educational (*B* = –0.58, 95% CI = [–0.79, –0.38], *p* < 0.001) and household income levels (*B* = –0.42, 95% CI = [–0.68, –0.15], *p* = 0.001). They also reported higher levels of depressed mood at baseline (*B* = 0.17, 95% CI = [0.01, 0.34], *p* = 0.038). Overall, this may suggest that there were some selection effects due to prior mental health status and socioeconomic background that influenced the likelihood of moving out or starting cohabitation relatively early in the life span. There was no indication of anticipatory effects on depressed mood directly prior to the actual transitions.

### 3.3 Parental SES as a moderator of the effects of early life transitions on depressed mood

In the multiple group model, the intercept of the growth model was allowed to vary freely across SES groups while the growth parameters were group invariant. We further assumed time-invariant effects across waves for the early life transitions in line with the findings from the previous paragraph. The estimates of the associations between early life transitions and depressed mood across SES groups are shown in Table 3. Evidence for moderation was found for parental educational level on leaving the parental home. The transition effect of moving out on depressed mood was most pronounced in the group with a high parental educational level. In this group, moving out was associated with a decrease in depressed mood. In the other two groups, the association with depressed mood was not statistically significant. Evidence for moderation was found for parental household income on leaving the educational system and attaining employment. Leaving the educational system was associated with lower depressed mood scores in the high-income group, while point estimates were opposite and non-significant in other the two income groups. Attaining employment was not statistically significant within any of the three income groups, but point estimates suggest that attaining employment may be associated with decreases in depressed mood in the low- and medium-income groups, while it may be associated with an increase in depressed mood in the high income group.

**Table 3:** Unstandardized estimates of life transitions on depressed mood across SES groups.

	**Low parental educational level**	**Medium parental educational level**	**High parental educational level**	**Wald test**
Leaving the parental home	−0.17 (−0.42, 0.08)	−0.04 (−0. = 17, 0.09)	**−0.31 (−0.44**, **−0.17)**	**χ**^**2**^ **=** **7.04, df** **=** **2**, ***p** **=*** **0.030**
Leaving the educational system	0.02 (−0.33, 0.36)	0.07 (−0.13, 0.26)	−0.07 (−0.27, 0.13)	χ^2^ = 0.987, df = 2, *p =* 0.611
Beginning cohabitation	**−0.35 (−0.48**, **−0.21)**	−0.12 (−0.26, 0.01)	0.00 (−0.18, 0.19)	χ^2^ = 1.126, df = 2, *p =* 0.570
Attaining employment	−0.07 (−0.46, 0.32)	−0.05 (−0.26, 0.16)	−0.08 (−0.32, 0.16)	χ^2^ = 0.042, df = 2, *p =* 0.979
	**Low household income level**	**Medium household income level**	**High household income level**	**Wald test**
Leaving the parental home	−0.16 (−0.40, 0.09)	**−0.17 (−0.30**, **−0.03)**	**−0.20 (−0.34**, **−0.04)**	χ^2^ = 0.97, df = 2, *p =* 0.953
Leaving the educational system	0.15 (−0.21, 0.51)	0.08 (−0.10, 0.25)	**−0.26 (−0.47**, **−0.05)**	**χ**^**2**^ **=** **8.09, df** **=** **2**, ***p** **=*** **0.018**
Beginning cohabitation	**−0.33 (−0.49**, **−0.18)**	−0.03 (−0.19, 0.12)	−0.13 (−0.31, 0.05)	χ^2^ = 0.724, df = 2, *p =* 0.70
Attaining employment	−0.24 (−0.64, 0.15)	−0.14 (−0.32, 0.04)	0.21 (−0.04, 0.46)	**χ**^**2**^ **=** **6.76, df** **=** **2**, ***p** **=*** **0.034**

Estimates in bold are significantly different from zero (*p* < 0.05).

## 4 Discussion

The present study addressed research questions pertaining to the development of depressed mood from adolescence to adulthood and to what extent parental education and household income are associated with differential exposure and development of depressed mood as conceptualized by mechanisms A and C in the APM. We also investigated if common early life transitions affect the development of depressed mood across time and whether socially differential vulnerability effects are indicated across levels of parental education and household income as suggested by mechanism D in the APM. Our findings showed that depressed mood peaked at age 15 and 18 and then followed a decreasing trend over time. Adolescents in the low parental education group had elevated depressed mood compared to the high group during early adolescence and mid-adulthood. Adolescents in the low household income group also showed increasing levels of depressed mood during young adulthood compared to the medium income group. For life transitions, leaving the parental home and beginning cohabitation were associated with a decrease in depressed mood whereas leaving school and becoming employed were not when adjusting for other transitions. Moderation by SES was observed with adolescents in the high parental education group showing a significant decrease in depressed mood when leaving the parental home compared to the medium and low parental education group. Adolescents in the high household income group also had a decrease in depressed mood when leaving the educational system compared to the low and medium groups. In contrast, attaining employment seemed to be associated with a decrease in depressed mood in the low and medium household income groups whereas the high household income group indicated an increase in depressed mood.

The overall decrease in depressed mood across time is akin to findings by Bracke et al. (64) who found a decline in depression among Norwegians aged 21 to 80 as measured on the CESD-8. However, since the present study started (1990), adolescents in Norway have increasingly reported health complaints (65), thus, our findings might differ from contemporary Norwegian adolescents. Nevertheless, the declining trajectory of depressive outcomes appear to be a shared feature of some Nordic countries where a small decline in depressive symptoms across time is indicated in Norway, Sweden and Denmark (64). However, for most other European countries, past adolescence, depressive symptoms seem to increase (64). Furthermore, we should note that females showed persistently higher levels of depressed mood from age 13 to age 40 - which aligns with other studies showing a significant gender gap in depressive outcomes (66, 67). This corroborates the common finding that gender is an important dimension of social inequality, which future studies should seek to address in conjunction with investigations of socioeconomic disparities in health.

Our findings also showed socially differential tracking of depressed mood by parental education and household income. A handful of studies have distinguished between indicators of parental SES, yet findings are too sparse to generalize about the relative strength of each. Our results indicate that household income might play a larger role in affecting offspring depressed mood during early adulthood (as opposed to mid-adulthood). The latter is supported by studies showing that parents with higher incomes tend to transfer more wealth to their offspring - especially during young adulthood (43, 44, 68). Earlier research further indicate that this is strongly linked to offspring' needs as they transition into and establish themselves as adults (i.e., financing daily living expenses, child care, economic security, housing etc.) (69). It could thus be that young adults coming from lower household incomes are more prone to depressive outcomes as their parents can't provide the same level of financial and material support as their higher SES counterparts - thereby providing less of a buffer effect on the stressors of adulthood (70, 71). However, it is noteworthy, that we did not find a significant difference in tracking of depressed mood between the low and high household groups. even though the latter group tend to receive higher levels of support (72). The findings could perhaps indicate that youth from higher household incomes are less dependent on parental households, but more research is warranted to investigate this further.

In contrast to household income, a US study from 2011 by McLaughlin et al. (73) found that parental education seems to play a more important role during early and mid adolescence when it comes to shaping offspring's mental health. More specifically, parental education seems to be linked to greater mental health literacy and access to mental health care services (73). However, a recent study of 10,257 Norwegian adolescents found little evidence of a parental education gap in access to and use of specialized mental health care (74). Still, the study by McLaughlin et al. (73) indicates that parental education is more closely associated with mood disorder severity whereas financial hardship is more associated with onset of mood disorder among adolescents. It thus may be that parental education matters more for depressed mood severity during adolescence when offspring are more dependent on their parents whereas parental education diminishes in importance as offspring become young adults and achieve greater psychological autonomy from parents while also increasingly turning to their spouse for support (75). From the present study, it is not clear why parental education is associated with a slight increase in depressed mood in mid-adulthood and whether this association sustains into older ages. It could be that offspring from more educated parental homes tend to have higher expectations or more pressure to attain higher status, however, more research is needed to empirically test these assumptions. In any case, both parental education and household income have been associated with a multitude of factors that might underpin these findings, warranting further research into the possible mechanisms at play.

Our findings on life transitions showed that changes in several of the living circumstances were associated with a decrease in depressed mood when adjusting for other life transitions. For leaving the parental home, this could partially be due to an improvement in the relationship with parents, e.g., being better able to relate responsibly and independently to parents which in turn has been associated with improved mental health as indicated by a longitudinal US study by Smetana et al. (76). Interestingly, in the present study, we also found that adolescents in the high parental education group seemed to have a higher decrease in depressed mood when moving out as compared to the medium and low group. This group of adolescents also tended to move out earlier than their peers. Together, these findings are similar to other studies which shows that higher SES adolescents are more motivated to move out and tend to do so earlier than their lower SES peers (77–80). This higher motivation to live on one's own could reflect the greater decrease in depressed mood experienced afterwards. More qualitative research could help shed light on why higher SES youth exhibit this motivation.

Like leaving the parental home, beginning cohabitation was also found to be associated with a drop in depressed mood. This could be due to higher levels of social support, a healthier lifestyle, and better economic resources which has been linked to being in a relationship as compared to being single (81, 82). This is also further supported by several studies which indicate that both marital and non-marital cohabitation is associated with better mental health than singlehood living (83). Though we did not find this transition to be moderated by either parental education nor household income, our findings did show that adolescents who were more depressed at baseline as well as coming from homes with lower parental education and household income were more likely to begin cohabitation early on. This was also found a meta-analysis across 25 European countries which showed that young adults from lower SES backgrounds initiated co-residential union earlier on than their higher SES counterparts (but with weaker associations in Nordic countries) (84). It has been suggested that this could be due to higher partner preferences (e.g., higher educational credentials and better income) among higher SES youth, but also parents expecting more thoughtful decisions in terms of partner choice (85, 86). Another factor could be that a negative family climate and conflictual relationships with parents (both of which are common among lower SES) are motivating factors for moving in with a partner early on (77, 87).

Leaving the educational system was associated with a drop in depressed mood when not controlling for other life transitions. In the adjusted analysis, this was also observed for the high household income group, whereas non-significant estimates for the low and medium household income groups indicated additive effects on depressed mood. Few similar studies have been done to compare with, however, studies from 1990 to 2010 comparing student and non-student mental health suggest significantly higher average depression prevalence of undergraduate university students than general and young adult population benchmark levels (9%−22%) (88). Furthermore, findings from the Health Behaviour in School-aged Children study (HBSC) reveal that lower SES youth tend to be more indifferent toward school and education as compared to higher SES youth, which lends support to our finding that higher household income youth experiencing a greater reduction in depressive outcomes when leaving secondary school (89).

Attaining employment was also associated with a drop in depressed mood when not controlling for other life transitions. This corresponds to a multitude of studies showing the beneficial effects of employment on depressive outcomes (47, 90). In addition, our findings paradoxically seem to indicate greater vulnerability for the high household income group of adolescents who showed higher depressed mood following attainment of full-time employment, however, none of the estimates were significant, and the transition did not exert a significant effect when controlling for other life transitions which could suggest that other transitions are more intimately tied to depressive trajectories.

All in all, though our findings do support socially differential exposure and tracking of depressed mood across time as indicated by the APM, we did not find clear evidence of socially differential vulnerability among lower SES youth. This could relate to national policies and the Nordic welfare system. For example, it could reflect the Norwegian welfare state acting as a buffer by providing a high level of income equality and easy access to a wide range of social welfare benefits, making, for instance, economic hardship less likely to affect depressed mood when leaving the parental home (91). Such an explanation was suggested in a Swedish study that looked at trajectories of education and labor market attachment from age 18 to 42 with depressive symptoms at age 16 as a predictor (92). No such association was found which the authors suggest might have to do with the buffering effect of the Swedish welfare state (92). However, other studies contrast these interpretations and suggest no effect of welfare systems on socioeconomic inequalities in health (93).

An additional explanation speaks to the general positive effects of transitioning into adulthood (35). That is, adolescents who succeed in leaving the parental home, finishing education, attaining full-time employment and beginning cohabitation can be said to succeed according to cultural expectations as well as individual psychological development (35). It is also possible that other life transitions or events would show more negative associations with depressive outcomes. For instance, unemployment in young adulthood has been linked to increased mental health problems (94). Similarly, several studies have computed indices a number of stressful life events (i.e., death of a parent, having a new partner moving into one's home, a romantic break-up etc.) and looked at their association with mental health outcomes (95). However, these studies have been criticized, because the life events that are aggregated vary greatly in emotional valence, in addition, the individual effects of each type of life event are not computed (95). Moreover, according to the life course literature, some transitions or events are considered more fundamental markers of adulthood than others (i.e., leaving the parental home, finishing school, finding a job, finding a partner etc.) (96). Still, more studies are needed to get a better empirical understanding of the impact of life transitions on depressive trajectories across different socioeconomic settings and whether disparities are more pronounced across societies and cultures.

### 4.1 Study strengths and limitations

To the authors' knowledge, no studies have employed a 27-year latent growth model to explore the effects of four major life transitions on depressed mood while also accounting for adolescent socioeconomic context and distinguishing between the effects of both parental education and income as individual indicators of SES. However, some limitations should be noted. Firstly, our study had low power to detect moderation by SES and based on the distributions of responses to the SES answer categories, we opted to use tertiles as cut-off points instead of answer categories (e.g., university degree vs. non-university degree). However, the estimates found in the current study could be useful in future meta-analyses. Secondly, the timing of life transitions and depressed mood within a given year was not evident from the sampling, thus, warranting caution in causal inferences. On a related note, we were not able to distinguish between students who graduated vs. dropped out when leaving the educational system - implying some ambiguity in this measure which may have affected the strength of its association with depressed mood. Thirdly, we had limited information on variables that could explain the identified associations between life transitions and depressed mood, such as family situation and relationship with parents prior to moving out (incl. reasons for the actual transitions). Such studies are needed to explore these mechanisms in greater depth. Fourthly, there has been limited use of our measure of depressed mood in the literature, and the relation to similar concepts, such as depressive symptoms and depression, is unclear. Still, our measure shows good concurrent validity with the CES-D measure of depressive symptoms. Finally, we should also mention that higher attrition at age 40 was associated with lower parental education, household income as well as being male and not becoming a parent at age 19 (See Appendix A). In addition, *post-hoc* analyses also showed parental education predicted leaving the parental home in 1995, and in addition, household income, parental education and baseline depressed mood predicted beginning cohabitation in 1996 - indicating some selection effects in our sample. Thus, our findings may be biased toward the inclusion of middle and higher SES individuals and individuals who became parents at an early age as well as containing some confounding for leaving the parental home and beginning cohabitation early in life.

## 5 Conclusions

Depressed mood decreased over time and developed differently depending on parental education and household income. Life transitions were generally associated with reductions in depressed mood across time, but lower SES youths were not found to be more socially vulnerable to the effects of these. This could indicate that life transitions generally have beneficial effects on depressed mood as they mark the transition into adulthood, however, it is noteworthy that depressed mood still showed clear social disparity from adolescence to mid-adulthood. Our findings point to the importance of studying life transitions along with other risks and protective factors to better understand how depressive trajectories unfold across time from different socioeconomic origins. Future research focusing on life transitions should ensure accurate temporal alignment between the exposure to life transitions and subsequent outcomes. It is also critical to adjust for relevant childhood confounders while incorporating measures that assess the emotional valence and rationale behind each studied life transition.

## Data availability statement

The original contributions presented in the study are included in the article/Supplementary material further inquiries can be directed to the corresponding author.

## Ethics statement

The studies involving humans were approved by the Regional Committee of Medical Research Ethics (REK). The studies were conducted in accordance with the local legislation and institutional requirements. Written informed consent for participation in this study was provided by the participants' legal guardians/next of kin.

## Author contributions

MJ: Formal analysis, Methodology, Writing—original draft, Writing—review & editing, Validation, Visualization. OS: Formal analysis, Methodology, Supervision, Writing—review & editing, Visualization. BW: Conceptualization, Funding acquisition, Project administration, Supervision, Writing—review & editing. EH: Project administration, Supervision, Writing—review & editing.
