# Supplementary material for: Social inequality in the association between life transitions into adulthood and depressed mood: a 27-year longitudinal study
Source: Front Public Health. 2024 Feb 27;12:1286554. doi: 10.3389/fpubh.2024.1286554 (PMC10929615; doi:10.3389/fpubh.2024.1286554)
Supplement: Supplementary file 1 [file Table_1.docx]

**Appendix A. Attrition analysis**

To investigate reasons for attrition in the study sample, we conducted independent sample t-tests and chi-square tests on the main study variables. We used data on student participation at age 40 (2017) as the grouping variable with 0 coded for non-participation and 1 coded for participation. Independent sample t-tests were done on parental education and household income as well as depressed mood at age 13 (See Table C1). Chi-square testing was done on gender and first-time occurrences for all life transitions variables (See Table C2). Results show that individuals with lower parental education and household income were more likely to have dropped out of the study at the final time point. Furthermore, a weak association was found for gender with more girls participating at follow-up than boys. For attaining full-time employment at age 18, we found that a weak association, but with uncertainty for the directionality as indicated by the opposing values of Phi and Cramer’s V. For parenthood, we found a strong positive association with young parents being more likely to participate at the final time point, although it should be mentioned that very few participants were parents at age 19.

**Table C1.** Independent sample t-tests for SES and depressed mood at age 13 for participation versus non-participation at age 40 (2017)

| Participation | Yes |  | No | |  |  | |  | |  | |  | | |  | |  |
| --- | --- | --- | --- | --- | --- | --- | --- | --- | --- | --- | --- | --- | --- | --- | --- | --- | --- |
|  | *n* | Mean  (SD) | *n* | Mean  (SD) | Mean difference | | F-value | | Two-tailed p-value | | | | t-value |  | | df | |
| Parental education | 429 | 2.77  (1.22) | 539 | 2.43  (1.22) | -.342 | | 8.304 | | < .001 | |  | | -4.533 | | | 966 | |
| Household income | 322 | 4.45  (1.20) | 293 | 4.12  (1.26) | -.327 | | .723 | | .001 | |  | | -3.304 | | | 613 | |
| Depressed mood at age 13 | 324 | 2.28  (.90) | 398 | 2.25  (.90) | -.025 | | .000 | | .716 | |  | | -.363 | | | 720 | |

**Table C2.** Chi-Square Test of Differences in participation versus non-participation at age 40 (2017) for gender and first-time occurrences of life transitions.

|  | *χ*^2^ | *df* | Two-tailed p-value | Phi | Cramer’s V |
| --- | --- | --- | --- | --- | --- |
| Gender | 3.949 | 1 | .047 | .056 | .056 |
| Leaving the parental home (age 18) | 2.436 | 1 | .119 | -.064 | .064 |
| Beginning cohabitation (age 19) | .512 | 1 | .474 | .028 | .028 |
| Leaving the educational system (age 16) | .632 | 1 | .427 | -.030 | .030 |
| Attaining full-time employment (age 18) | 6.033 | 1 | .014 | -.088 | .088 |
| Parenthood (age 19) | 20.092 | 1 | .000 | .176 | .176 |
