# Supplementary material for: Social inequality in the association between life transitions into adulthood and depressed mood: a 27-year longitudinal study
Source: Front Public Health. 2024 Feb 27;12:1286554. doi: 10.3389/fpubh.2024.1286554 (PMC10929615; doi:10.3389/fpubh.2024.1286554)
Supplement: Supplementary file 2 [file Table_2.docx]

**Appendix B. Measurement Invariance**

Due to similar wording, residual correlations between item 3 and 5 (“I am often sad without seeing any reason for it” and “I often feel down without knowing why”) were allowed to covary. Partial scalar invariance was achieved by freeing the intercepts of item 1 in 2007 and 2017, item 2 and 5 in 1993, item 3 in 1991 and item 7 in 2017. All intercepts for item 4 and 6 remained intact. The final model yielded acceptable fit indices (see table below).

**Table B1. Longitudinal measurement invariance**

|  | *χ*^2^ | *df* | RMSEA [90%CI] | CFI | SRMR | ΔRMSEA | ΔCFI | ΔSRMR |
| --- | --- | --- | --- | --- | --- | --- | --- | --- |
| **Longitudinal** |  |  |  |  |  |  |  |  |
| Configural | 4002.424 | 2077 | .029 [.028,.030] | .923 | .052 |  |  |  |
| Metric | 3739.842 | 2029 | .028 [.026,.029] | .932 | .052 | .001 | .009 | .000 |
| Scalar | 4314.980 | 2083 | .031 [.030,.033] | .911 | .053 | .003 | .021 | .001 |
| Partial Scalar | 4002.424 | 2077 | .029 [.028,.030] | .923 | .052 | .002 | .012 | .001 |

Note. Χ^2^ = chi square; df = degrees of freedom; RMSEA = the root mean square error of approximation; CI = confidence interval; CFI = comparative fit index; SRMR = standardized root mean square residual.

Because of model estimation difficulties for group invariance (low covariance coverage), we chose three time points to test measurement invariance across - corresponding to baseline (1990), mid-point (1996) and final time point (2017). Residual correlations between item 3 and 5 as well as item 4 (“Sometimes I think everying is so hopeless that I don’t feel like doing anything”) and 5 were added to the configural model. For parental education our SRMR slightly exceeded the recommended cut-off of ≥ .08, but CFI and RMSEA showed good fit, and we thus considered scalar invariance achieved across parental education groups. Parental income also showed high levels of SRMR, yet as CFI and RMSEA worked as our primary fit indices, we considered scalar invariance achieved across these groups as well. This process was done in accordance with recommendations by Chen (1) who writes on p. 501: “Among the three indexes, CFI was chosen as the main criterion because RMSEA and SRMR tend to over- reject an invariant model when sample size is small, particularly when using SRMR for testing loading or residual variance invariance”.

**Table B2. Measurement invariance across parental education groups**

|  | *χ*^2^ | *df* | RMSEA [90%CI] | CFI | SRMR | ΔRMSEA | ΔCFI | ΔSRMR |
| --- | --- | --- | --- | --- | --- | --- | --- | --- |
| Configural | 712.459 | 441 | .046 [.040,.052] | .950 | .075 |  |  |  |
| Metric | 736.099 | 477 | .043 [.037,.049] | .952 | .081 | .003 | .002 | .006 |
| Scalar | 778.816 | 513 | .042 [.036,.048] | .951 | .081 | .001 | .001 | .001 |

Note. Χ^2^ = chi square; df = degrees of freedom; RMSEA = the root mean square error of approximation; CI = confidence interval; CFI = comparative fit index; SRMR = standardized root mean square residual.

**Table B3. Measurement invariance across parental income groups**

|  | *χ*^2^ | *df* | RMSEA [90%CI] | CFI | SRMR | ΔRMSEA | ΔCFI | ΔSRMR |
| --- | --- | --- | --- | --- | --- | --- | --- | --- |
| Configural | 711.426 | 441 | .056 [.048, .064] | .938 | .083 |  |  |  |
| Metric | 749.090 | 477 | .054 [.047,. 061] | .938 | .090 | .002 | .000 | .007 |
| Scalar | 803.115 | 513 | .054 [.047, .061] | .934 | .090 | .000 | .004 | .000 |

Note. Χ^2^ = chi square; df = degrees of freedom; RMSEA = the root mean square error of approximation; CI = confidence interval; CFI = comparative fit index; SRMR = standardized root mean square residual.

1. Chen FF. Sensitivity of Goodness of Fit Indexes to Lack of Measurement Invariance. Structural equation modeling. 2007;14(3):464-504.
